# Beyond genetics: integrative oncology and the metabolic perspective on cancer treatment

**DOI:** 10.3389/fonc.2024.1455022

**Published:** 2024-09-18

**Authors:** Pradeep MK Nair, Karishma Silwal, Renganathan Ramalakshmi, Muniappan Devibala, Maruthanayagam Saranya, Sekar Sivaranjani, Thangavelu Ramasamy, Ayyappan Palanisamy, Manickam Mahalingam

**Affiliations:** ^1^ Department of Integrative Oncology, Mirakle Integrated Health Centre, Pollachi, India; ^2^ Department of Naturopathy, Sant Hirdaram Medical College of Naturopathy and Yogic Sciences, Bhopal, India

**Keywords:** metabolism, Warburg theory, complementary medicine, metabolic disease, cancer

## Abstract

Cancer is traditionally approached as a genetic disease, with standard treatments including chemotherapy, radiation, targeted therapy, immunotherapy, and surgery significantly improving survival rates and patient outcomes. However, there is a growing recognition of the need for integrative oncology, which expands cancer management by considering cancer as a metabolic disease. Integrative medicine physicians employ holistic therapies focused on patients’ needs, aiming to correct the metabolic imbalances associated with cancer and alleviate cancer-related symptoms. Viewing cancer as a metabolic disease involves addressing factors such as an acidic microenvironment, vitamin C deficiency, mitochondrial dysfunction, reduced intracellular oxygen levels, elevated oxidative stress, dysfunctional autophagy, and psychological stress. This paper presents an overview of the evidence and comprehensive strategies supporting integrative medicine approaches in addressing cancer metabolism in integrative oncology settings. Furthermore, the paper underscores the necessity of integrating different cancer theories—genetic and metabolic—for improved patient outcomes and experiences. By combining these perspectives, integrative oncology offers a more holistic, patient-centered approach to cancer treatment.

## Introduction

Cancer is a complex disease that poses multiple challenges to patients and their caregivers. Emerging evidence suggests that cancer is a metabolic disease resulting from mitochondrial dysfunction and impairment in energy metabolism (1, 2). Further, the discovery of oncometabolites like fumarate, sarcosine, glycine, asparagine, choline, lactate, glucose, glutamine, and serine which are usually found increased in quantity in cancer to support the aerobic glycolysis, glutaminolysis, and one-carbon metabolism has strengthened the approach of treating cancer as a metabolic disease (3). However, present-day cancer management strategies do not largely consider this and treat cancer as a genetic disorder. While conventional therapies like chemotherapy, radiation, surgery, and immunotherapy remain the gold standard in the management of cancer, integrative medicine approaches that include yoga, biologicals, nutritional therapies, ozone therapy, and acupuncture are also increasingly becoming popular among cancer patients and physicians. This approach is popularly known as integrative oncology (4, 5). Integrative medicine physicians use these therapies holistically, focused on the patient’s needs, and aimed at correcting the metabolic disarrays associated with cancer as well as reducing the symptoms associated with cancer.

Cancer development and progression are intricately linked to various metabolic dysfunctions. These metabolic disturbances are thought to precede the genetic alterations that are considered the primary causes of cancer development (2). Addressing these metabolic dysfunctions through integrative medicine approaches offers potential therapeutic benefits for cancer management. It is very important to emphasize the fact that integrative medicine therapies do not replace conventional cancer treatments but rather complement them. By addressing the metabolic and psychosocial aspects of cancer, integrative therapies can enhance the efficacy of standard treatments, reduce side effects, and improve overall patient outcomes.

**Figure 1:**
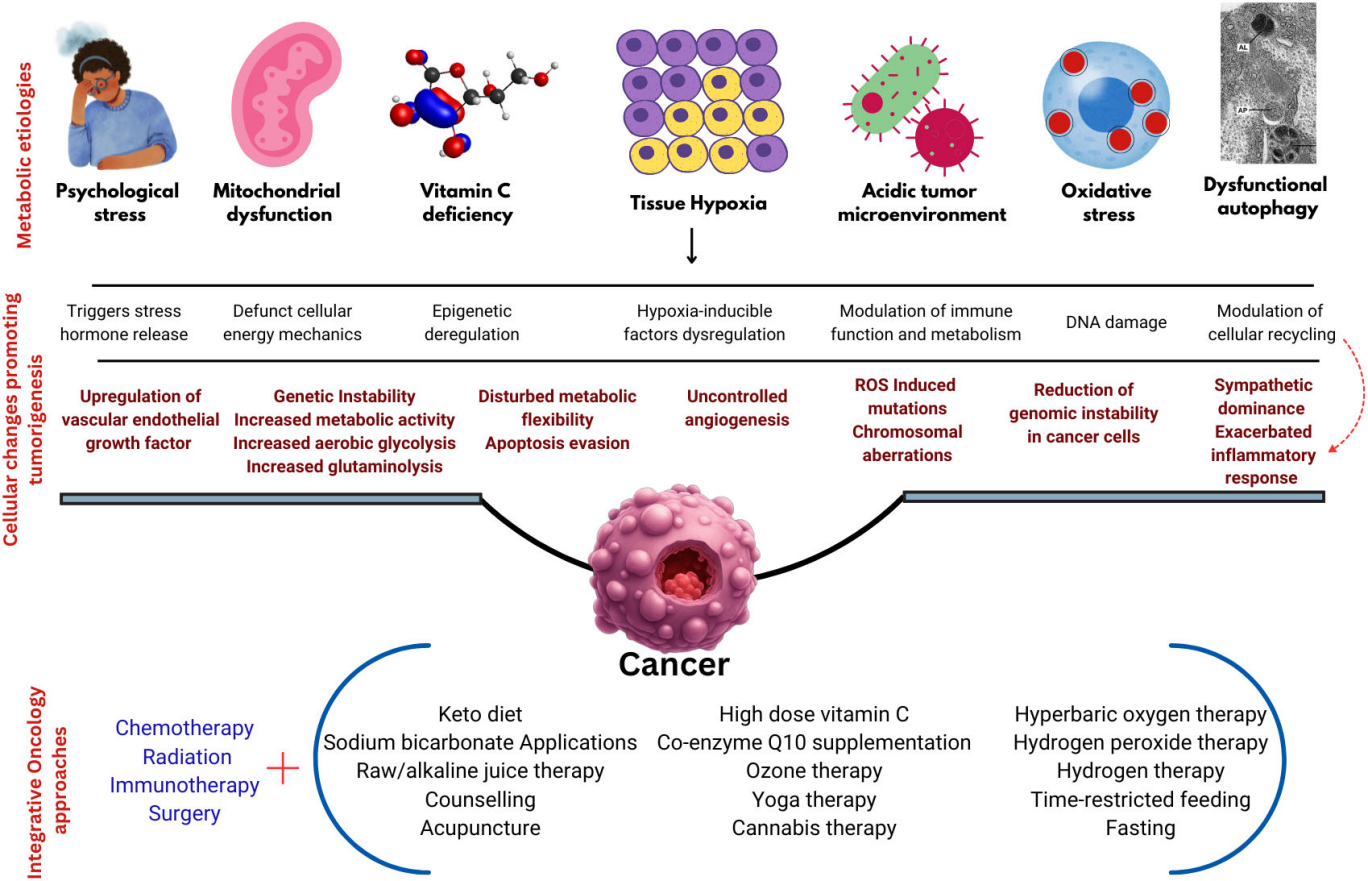
The metabolic causation of cancer and proposed integrative oncology approaches.

This paper provides an overview of such approaches, targeting metabolic dysfunctions like elevated acidic microenvironments, vitamin C deficiency, dysfunctional mitochondria, reduced intracellular oxygen levels, increased oxidative stress, impaired autophagy, and psychological stress. Further, this paper also attempts to provide the rationale behind the use of various integrative medicine therapies and their practical methodologies. **Figure 1** illustrates the metabolic causation of cancer and the proposed integrative oncology approaches.

## Acidic microenvironment and cancer

The acid-base status of normal tissues ranges between 7.3 to 7.4 under optimal conditions. However, the tumor microenvironment is highly acidic due to derailed metabolism, rapid cell division, and hypoxia. This extracellular acidic microenvironment is thought to support the development and progression of cancer cells (6). Studies report acidic environment leads to genetic instability, (7) delay in DNA repair, (8) increased metabolic activity, (9) reduced glycolytic activity, immune evasion, (6) accelerated conversion of glutamine to glutamate, (10) and increased chances of metastases (6, 11).

Altering the acidic extracellular milieu may have great therapeutic potential in addressing cancer cell metabolism (12). Numerous *in vitro* and vivo studies have shown the use of an alkaline environment (sodium bicarbonate-NaHCO3, dietary modification) to inhibit cancer growth, prevent metastases, and increase the survival rate (13, 14). Integrative medicine physicians use sodium bicarbonate in the form of oral drinks, nebulization, and gargling. Besides this, the patients are also advised to follow an alkaline juice diet of raw fruit and vegetable juices. Both these approaches are aimed at improving the alkalinity in the tissues and preventing the progression of cancers. **Supplementary File 1** provides a detailed practical methodological explanation of the therapies discussed above.

## Vitamin C and cancer

Vitamin C is an important micronutrient and metabolic regulator, which plays an active role in various metabolic and immune-related functions such as glycolysis, cell division, and redox balance (15). Furthermore, vitamin C also functions as an epigenetic modulator, decreasing the risk of cancer development and progression (16). Several studies highlight an inverse association between vitamin C and cancer, reporting low vitamin C levels among cancer patients (17–19). A recent umbrella review on vitamin C and cancer has reported that vitamin C consumption lowers the incidence of cancers in the bladder, breast, cervix, endometrium, esophagus, stomach, brain, lungs, pancreas, prostates, and kidneys (20). Humans cannot produce vitamin C, so it must be obtained from external sources. The use of vitamin C in cancer treatment is a double-edged sword, requiring careful dosage management. At low doses, it functions as an antioxidant, but at high doses, it acts as a pro-oxidant. As a pro-oxidant, Vitamin C induces the production of hydrogen peroxide, disrupting the cellular redox balance in cancer cells leading to cell death (21).

High dose vitamin C as a potential anti-cancer therapy is increasingly becoming popular among integrative oncologists. A recent overview encapsulated the anticancer impact of vitamin C as eliciting oxidative damage, regulating epigenetic processes, dampening adaptive responses to hypoxia, and facilitating the synthesis of collagen and neurotransmitters (22). In integrative medicine settings, intravenous and oral high-dose vitamin C therapy are provided for cancer patients. The usual doses are up to 1.5 gm per kg of body weight for all the patients, as per standard recommendations from earlier studies (23). The detailed practical methodological explanation for vitamin C administration is provided in **Supplementary File 1**.

## Mitochondria and cancer

The concept of cancer as a mitochondrial disease is attracting increased attention, thereby shifting its characterization from solely a genetic disorder to a metabolic one (24). The relationship between metabolic dysregulation and mitochondrial dysfunction in cancer is complex and often interdependent. Beyond bioenergetics, mitochondria play multiple roles inside a cell such as contributing to cellular transformation, encompassing processes such as modulation of fission and fusion, promotion of natural cellular apoptosis, regulation of oxidative stress, metabolic activities, and signaling pathways (25). Seyfried and colleagues, who have conducted extensive research on this topic, suggest that a metabolic approach in cancer management could significantly impact cancer treatment by targeting metabolic dysfunction in mitochondria. This involves shifting from fermentable metabolites to fats or ketone bodies, which possess anti-angiogenic, anti-inflammatory, and pro-apoptotic properties in tumor cells (26). Furthermore, Warburg’s work emphasizes the role of mitochondria in tumorigenesis, primarily due to impaired respiration at the mitochondrial level, which fails to inhibit oncogene expression promoting tumorigenicity (27, 28).

Mitochondrial dysfunction induces the metabolic inflexibility of cancer cells by dependence on fermentable fuels, which supports them to thrive in various microenvironments, especially under nutrient and oxygen limitations, but makes them vulnerable to metabolic targeting (29). Further, mitochondrial dysfunction is intricately linked to cancer development and progression through various mechanisms, including mtDNA mutations, ROS production, apoptosis evasion, and metabolic reprogramming. Given the central role of mitochondria in cancer, they are attractive targets for therapy. Therefore, targeting this altered metabolism is a potential therapeutic strategy in cancer treatment.

In integrative medicine settings, a combination of a keto diet, coenzyme Q supplementation, and ozone therapy is used as the primary approach to tackle mitochondrial dysfunction. Studies recommend the keto diet as a potential therapy to overcome mitochondrial dysfunction. The keto diet impacts mitochondrial dynamics, mitophagy, and mitochondrial redox metabolism which helps in repairing mitochondrial dysfunction (30). Co-enzyme Q 10 supplementation is another strategy adapted to overcome mitochondrial dysfunction. Research indicates that co-enzyme Q10 supplementation activates mechanisms that regulate mitochondrial biogenesis (31). Ozone therapy has been demonstrated to upregulate mitochondrial dynamics, thereby mitigating mitochondrial dysfunction. The mechanisms through which ozone therapy enhances mitochondrial function include improved oxygen utilization, modulation of oxidative stress, stimulation of mitochondrial biogenesis, and enhancement of mitochondrial dynamics (32, 33). **Supplementary Table 1** outlines the practical methodological explanation for all the therapies discussed in this section.

## Intracellular oxygen levels and cancer

Oxygen is essential for cellular metabolism and function, and intracellular oxygen levels play a crucial role in maintaining cellular homeostasis and influencing disease states, including cancer. Hypoxia-inducible factors (HIFs) are key transcriptional regulators activated in low-oxygen conditions. HIF-1α and HIF-2α stabilize and move to the nucleus, where they stimulate the expression of genes involved in angiogenesis, metabolism, cell survival, and invasion (34). Additionally, to counteract hypoxia, tumor cells induce angiogenesis, the formation of new blood vessels, through the upregulation of vascular endothelial growth factor (VEGF) and other pro-angiogenic factors (35, 36). Cancer cells adapt by shifting their energy production from oxidative phosphorylation to glycolysis, a phenomenon known as the Warburg effect. This metabolic shift allows cancer cells to thrive in low-oxygen environments, supports rapid cell growth, and contributes to the acidic tumor microenvironment, promoting invasion and metastasis (28).

Hyperoxygenation of cancer cells through ozone therapy and hyperbaric oxygen therapies are thought to have inhibitory effects on cancer cell metabolism (37). Hyperbaric oxygen therapy has been reported to have an inhibitory effect on the expressions of HIF-1α, which in turn exhibits an anti-angiogenic effect. This reduction in angiogenesis can starve tumors of the necessary blood supply, thereby inhibiting their growth and ability to metastasize (38). Further, elevated oxygen levels can increase the production of reactive oxygen species (ROS) within cancer cells. While moderate levels of ROS are involved in cell signaling and survival, excessive ROS can cause oxidative damage to cellular components, favoring cancer cell apoptosis and anti-tumorigenesis (37, 38). In summary, by inhibiting HIF-1α, reducing angiogenesis, and increasing oxidative stress within cancer cells, both hyperbaric oxygen and ozone therapy can complement traditional cancer treatments and improve patient outcomes. In an integrative medicine setting, ozone is used in various forms, including major autohemotherapy, minor autohemotherapy, rectal insufflation, ear insufflation, ozone bagging, hyperbaric ozone, and breathing ozone through oil. Refer to **Supplementary File 1** for detailed practical methodologies of the therapies discussed.

## Oxidative stress and cancer

Metabolic dysfunction and oxidative stress are central features of cancer cell biology, driving tumor growth and progression. Metabolic dysfunction can lead to oxidative stress through various interconnected mechanisms such as increased ROS production, mitochondrial dysfunction, and impaired antioxidant defense mechanisms (39). This interplay contributes to cellular damage and is a key factor in the development and progression of diseases including cancer.

Oxidative stress plays a dual role in cancer, contributing to both the initiation and progression of the disease, as well as serving as a target for therapeutic intervention. Oxidative stress can cause significant DNA damage, resulting in mutations, strand breaks, and chromosomal aberrations. This DNA damage is a critical factor in the initiation of cancer, as it can lead to genetic instability and the activation of oncogenes or the inactivation of tumor suppressor genes. Oxidative stress, resulting from metabolic dysfunction, can induce mutations that contribute to the transformation of normal cells into cancerous cells (39, 40). Additionally, ROS can modulate various cell signaling pathways involved in cell proliferation, apoptosis, and differentiation. Chronic activation of these pathways by oxidative stress can promote tumor growth and survival (40). Oxidative stress can further catalyze the cancer progression by altering the tumor microenvironment, (41) inducing epithelial-mesenchymal transition, (42) and contributing to the immune evasion of cancer cells (40).

Given the role of oxidative stress in cancer causation, antioxidant therapy has been proposed as a potential strategy for cancer prevention. Antioxidants such as vitamins C and E, N-acetylcysteine (NAC), and polyphenols can neutralize ROS and reduce oxidative damage. However, the efficacy of antioxidant therapy in cancer treatment remains controversial. Some studies suggest that antioxidants may protect cancer cells from ROS-induced apoptosis, potentially promoting tumor survival (41, 43). This indicates the logical need for strategies that further enhance oxidative stress within cancer cells to induce cancer cell death while protecting normal cells.

In integrative medicine settings, a pro-oxidant approach is often used, aiming to increase ROS levels to a toxic threshold that selectively kills cancer cells, which lack anti-oxidative defense mechanisms, while sparing normal cells that can resist oxidative damage. Hydrogen peroxide inhalation, (44) hydrogenated water drinking, (45) and oral or intravenous high dose vitamin C, (21) are the common pro-oxidant therapies used. Nevertheless, patients are advised to follow an antioxidant-rich diet plan that includes millets, leafy vegetables, soups, and seasonal fruits, aimed at reducing metabolic dysfunctions. Refer to **Supplementary File 1** for detailed practical methodology of the therapies discussed.

## Dysfunctional autophagy and cancer

Similar to oxidative stress, autophagy plays a dual role in cancer by acting as a tumor suppressor in early stage tumors but as a survival mechanism for established tumors. In the context of tumor suppression, autophagy prevents the accumulation of damaged organelles and proteins, reducing oxidative stress and genomic instability. Loss of key autophagy genes, such as Beclin-1 (BECN1), has been associated with increased tumorigenesis in various cancer models (46). Given its dual role, targeting autophagy in cancer therapy requires a nuanced approach. Inhibiting autophagy could enhance the effectiveness of chemotherapy and radiotherapy by preventing cancer cells from recycling damaged components and surviving treatment-induced stress. Autophagy inhibitors, such as chloroquine and hydroxychloroquine, have been studied in combination with conventional therapies to improve treatment outcomes (47).

Conversely, in certain contexts, inducing autophagy could be beneficial, particularly in early-stage cancers or cancers with defective autophagy machinery (48). In this scenario, autophagy plays a crucial role in tumor suppression by maintaining cellular homeostasis, reducing oxidative stress, and preventing the accumulation of damaged proteins and organelles. It can inhibit the early stages of tumorigenesis by degrading oncogenic proteins and reducing DNA damage (46). In integrative medicine settings, fasting and time-restricted feeding (TRF) are two strategies used to address metabolic pathways and regulate autophagy. During fasting, nutrient-sensing pathways such as the insulin/IGF-1 and mechanistic target of rapamycin (mTOR) pathways are downregulated, leading to the activation of autophagy (49). TRF is a dietary regimen that restricts food intake to specific windows of time, typically 6-10 hours, without reducing overall caloric intake. This eating pattern synchronizes feeding with the body’s circadian rhythms, enhancing metabolic health and inducing autophagy similar to fasting by downregulating mTOR and activating activated protein kinase(AMPK) pathways (50).

Given the dual role of autophagy in cancer, personalized approaches are essential. Individual variations in response to fasting and TRF should be considered to optimize treatment plans (51).

## Psychological stress and cancer

Stress is considered one of the predisposing as well as progressive etiological factors for many diseases including cancer. However, most of the time this factor is not considered while planning treatment strategies for cancer. Psychological stress has been shown to influence mitochondrial function in multiple ways which has a direct impact on metabolic and immune regulation (52). Furthermore, psychological stress triggers the activation of the hypothalamic-pituitary-adrenal (HPA) axis and the sympathetic nervous system (SNS). This activation results in the release of stress hormones such as cortisol, adrenaline, and noradrenaline. These hormones can influence various physiological processes, including immune function, inflammation, and cellular metabolism, all of which are implicated in favoring tumor microenvironment (53). Chronic stress can suppress the immune system by reducing the activity and efficacy of immune cells such as natural killer (NK) cells and cytotoxic T lymphocytes. These cells play a crucial role in identifying and destroying cancer cells. Impaired immune surveillance due to stress can lead to increased tumor growth and metastasis (54). Stress hormones can promote angiogenesis by upregulating VEGF, which supports tumor growth and metastasis by enhancing blood supply and pathways for cancer spread (55).

Interventions like cognitive-behavioral therapy (CBT), mindfulness-based stress reduction (MBSR), and physical exercise show promise in improving the quality of life for cancer patients by reducing psychological stress and its physiological impact, potentially influencing cancer progression (56). In integrative medicine settings, yoga therapy, acupuncture, medical cannabis, mud packs, laser therapy, and counselling are used to deal with the psychological stress of cancer patients. These therapies aim to attenuate the stress response and address metabolic dysfunctions. Yoga therapy has been shown to enhance mitochondrial health, which is crucial for maintaining metabolic balance (57, 58). Acupuncture helps regulate energy flow, attenuates mitochondrial dysfunction, modulate energy metabolism, and improve overall metabolic processes (59). Medical cannabis interacts with the endocannabinoid system to modulate the mitochondrial dynamics, aiding in metabolic regulation (60). Mud therapy is known for its neuroendocrine-immune regulation, through which it downregulates inflammation and upregulates metabolic functions (61). Laser therapy enhances mood, improves mitochondrial function, promotes tissue repair, and reduces oxidative stress, thereby enhancing metabolic function (62, 63). Counselling encourages healthy lifestyle changes and reduces stress responses, contributing to better metabolic function (64).

## Discussion

Cancer is predominantly approached as a genetic disease, with standard treatments including chemotherapy, radiation, targeted therapy, immunotherapy, and surgery. While these conventional methods have undeniably improved survival rates and patient outcomes, there is a growing recognition of the need for integrative oncology which broadens cancer management by incorporating a metabolic perspective.

Integrative oncology combines traditional treatments with complementary therapies that target the metabolic aspects of cancer. This holistic approach addresses the underlying metabolic dysregulation in cancer cells, offering new avenues for treatment. For example, dietary interventions such as fasting and time-restricted feeding can induce autophagy, potentially enhancing the effectiveness of conventional therapies. Additionally, ozone therapy and high-dose vitamin C are emerging as potential adjunct treatments. Ozone therapy is thought to upregulate mitochondrial dynamics and improve cellular oxygen utilization, while high-dose vitamin C is believed to exert pro-oxidant effects selectively toxic to cancer cells. Moreover, managing psychological stress through techniques like mindfulness, cognitive-behavioral therapy, and physical exercise can enhance immunosurveillance and reduce inflammation, thereby potentially inhibiting tumor growth and improving overall patient well-being.

Despite the promise of these integrative approaches, it is crucial to acknowledge the current lack of robust evidence supporting their widespread use (refer **Supplementary File 1**). Many of these therapies, including ozone therapy and high-dose vitamin C, require more extensive clinical research to validate their efficacy and safety. Larger, well-designed studies are necessary to establish definitive evidence and integrate these treatments into standard oncology practice confidently. Nevertheless, this perspective in cancer management broadens the understanding of cancer and suggests additional ideas on avenues for treatment.

## Conclusion

Cancer is a multi-dimensional disease which requires pluralistic therapeutic approaches. The incorporation of metabolic therapies, stress management, and other integrative practices into cancer treatment protocols not only aims to improve survival but also enhances the quality of life for cancer patients. This multifaceted strategy underscores the importance of personalized medicine, where treatment is tailored to the individual’s unique genetic, metabolic, and psychosocial profile. By integrating these diverse approaches, the field of oncology can move towards a more comprehensive and effective management of cancer, ultimately leading to better outcomes and improved patient experiences.

## Data Availability

The original contributions presented in the study are included in the article/**Supplementary Material**. Further inquiries can be directed to the corresponding author.
