# Supplementary material for: Beyond genetics: integrative oncology and the metabolic perspective on cancer treatment
Source: Front Oncol. 2024 Sep 18;14:1455022. doi: 10.3389/fonc.2024.1455022 (PMC11456992; doi:10.3389/fonc.2024.1455022)
Supplement: Supplementary file 1 [file Table1.docx]

**Supplemental file 1: Practical methodology of metabolic therapies used by integrative physicians**

| **Treatments** | **Route of administration** | **Techniques** | **Authors** | **Study Design**** | **Key Findings** | **Level of evidence** and remarks** |
| --- | --- | --- | --- | --- | --- | --- |
| Sodium bicarbonate applications (NaHCO3) | Oral | 1 Teaspoon of NaHCO3 mixed in 500 ml of water | Estrella V et al^1^  Robey IF et al^2^  Robey IF et al^3^  Pötzl J et al^4^  Raghunand N et al^5^  Faes S et al^6^  Pilon-Thomas S et al^7^  Abumanhal-Masarweh H et al^8^  Takeda Y et al^9^  Yamazaki K^10^  Ding B et al^11^  Gillies RJ et al^12^ | Preclinical  Preclinical  Preclinical  Preclinical  Preclinical  Preclinical  Preclinical  RCT  NRCT  Preclinical  RCT | NaHCO3 inhibits tumor growth by raising the peritumoral pH.^1^  Reduce tumor metastases^2^  Inhibit the tumor invasion and increase the survival period.^3^  NaHCO3 upregulate natural killer (NK) cells function by which it delays tumor growth.^4^  NaHCO3 enhances the effectiveness of chemotherapy.^5^  Reduces the tumor angiogenesis.^6^  NaHCO3 combined with immunotherapy had modest or no effect on tumor growth.^7^  NaHCO3 exhibit anti-carcinogenic activity and improves the efficacy of cancer drugs.^8^  NaHCO3 does not improve the efficacy of chemotherapy drugs.^9,10^  NaHCO3 alkalizes the tumor microenvironment, regulates lactic acid metabolism, and enhances the effects of immunotherapy.^11^  No significant anti-carcinogenic effect except reduction in cancer-related pain.^12^ | **Level C (II)**  The clinical trials have smaller sample size and majority of the evidence are from in vitro or vivo studies. |
|  | Nasal | 1 ml of NaHCO3 with 4 ml of normal saline (NS) over 15 minutes |  |  |  |  |
|  | Intravenous | 50 ml of NaHCO3 with 30 ml of vitamin C in 450ml of NS administered over 90 minutes |  |  |  |  |
| Vitamin C | Oral^ꭍ^ | Liposomal vitamin C drink (200 ml) taken 3-4 times a day. | Fritz H et al^13^  Sebastian S et al^14^  Polireddy K et al.^15^  Mohseni S et al^16^  Nielsen TK et al.^17^  Hoffer LJ et al^18^  Schoenfeld JD et al^19^  Ou J et al.^20^  Ou J et al.^21^ | SR  NRCT  RCT  SR  CT  RCT RCT  RCT  RCT | High dose Vitamin C(HVC) tumor mass and improve survival in combination with chemotherapy.^13^  HVC exerts anti-inflammatory effects on cancer cells by reducing levels of CRP and ESR.^14^  Depletion of cellular NAD+ in cancer cells, as opposed to normal cells, leads to reduced ATP levels and significantly increases α-tubulin acetylation in cancer cells.^15^  HVC may have positive effects on cancer survival.^16^  No significant effect on cancer cells.^17^  HVC enhances the impact of chemotherapy and facilitates disease control and remission.^18, 19^  HVC improves quality of life and survival rate when combined with complementary therapies.^20,21^ | **Level B (I)**  Even though RCTs suggest the efficacy of vitamin C in cancers, systematic reviews indicate inconclusive evidence for making a recommendation and call for further robust trials. |
|  | Intravenous | 7.5 to 100 gms of vitamin C (depends on weight of patient ie.,1.5gms/kg of weight) |  |  |  |  |
| CO-Q 10 enzyme | Oral^ꭍ^ | 25mgs/day | Roffe L et al^22^  Dadali T et al^23^  Hu C et al^24^  Tafazoli A^25^  Fouad AA et al^26^  Abdel‐Latif et al^27^  Frontiñán-Rubio J et al^28^  Lockwood K et al^29^ | SR  Preclinical  Preclinical  SR  Preclinical  Preclinical  Preclinical  CT | Coenzyme Q10 (CoQ10) may help in improving the cancer treatment outcomes.^22^  Delivering oxidized Coenzyme Q10 to boost the mitochondrial Q-pool increases ROS, enhancing anti-cancer effects.^23^  CoQ10 exhibited an inhibitory effect on cell proliferation, as well as on migration and invasion of cancer cells.^24^  Coenzyme Q10 can target the mechanisms underlying breast cancer tumor progression.^25^  CoQ10 reduces lipid peroxidation, maintains glutathione and superoxide dismutase activity, and decreases tumor necrosis factor-α and nitric oxide levels.^26^  CoQ10 reduces cell proliferation, histological changes, AFP and TNF-α levels in hepatocellular carcinoma by altering lipids, CD59 expression, and phospholipase D activity.^27^  CoQ10 modulates the angiogenesis, and monocyte infiltration.^28^  CoQ10 offers tumor regression in breast cancer.^29^ | **Level C (II)**  Although there are systematic reviews suggesting the usefulness of CoQ 10 in cancers, all the systematic reviews discusses evidence from preclinical studies, except for one clinical trial. |
| Keto Diet |  | The ketogenic (keto) diet is a high-fat, low-carbohydrate diet, where carbs are limited to about 5-10% of daily caloric intake, 70-75% of daily calories come from fats, and 20-25% of daily calories from protein. | Zhao H et al^30^  Romer M et al^31^  Yang YF et al^32^  Klement RJ et al^33^  Klement RJ et al^34^ | SR & MA  SR  SR & MA  RCT  RCT | There were no significant changes in IGF-1 and TNF-α related to tumor growth.^30^  No conclusive evidence for anti-tumor effects.^31^  Inadequate evidence to support the beneficial effects of keto diet on antitumor therapy.^32^  The keto diet influences several metabolic health biomarkers, including lowering gamma-glutamyl-transpeptidase (GGT) and the triglyceride-glucose index, while improving the HDL cholesterol/triglyceride ratio and free T3 levels.^33^  Improvement in metabolic parameters such as gamma-glutamyl-transpeptidase (GGT) and the triglyceride-glucose index.^34^ | **Level C (I)**  Current studies suggest that the keto diet is a promising intervention for modulating the tumor microenvironment. However, considering the inconclusive evidence suggested by the meta-analysis and systematic reviews, more research with larger sample sizes and different cancer types is needed. |
| **Ozone Therapy**  Major Autohaemotherapy  Minor Autohaemotherapy  Rectal insufflation  Ear insufflation  Ozone Bagging | Intravenous  Intramuscular  Rectal  Otic  Topical  Intravenous  Nasal | Ozone concentration used in this procedure is between 20-40 μg/ml and 200 ml of blood is mixed with equal quantity of ozone oxygen mixture.  In this procedure, ozone is mixed with 2-3 ml of blood and is mixed with 5ml of ozone at 20-30 μg/ml concentration and injected intramuscularly.  Ozone oxygen mixture 10-40 μg/ml concentration is introduced through rectum via a catheter.  Ozone is introduced through the ear at a concentration varying from 10-30 μg/ml over 5 minutes.  The patient’s body is exposed to ozone after fully covering the desired body part with a polythene cover. The dose concentration ranges from 10 to 60 μg/ml.  Up to 2 litres of the patient's blood is drawn using sterile techniques. An anticoagulant is added to the blood to prevent clotting during the procedure. The drawn blood is mixed with an equal volume of the ozone-oxygen mixture (10 to 60 μg/ml concentration). This process is performed using specialized equipment to ensure thorough and sterile mixing. The ozonated blood is then slowly reinfused back into the patient.  Ozone at a concentration of 15 μg/ml is bubbled in a vegetable oil and then through a nasal cannula over 15 minutes. | Baeza-Noci J et al^35^  Clavo B et al^36^  Clavo B et al^37^  Yıldırım M et al^38^  König B et al^39^  Tang S et al^40^  Simonetti V et al^41^ | SR  CT  Review  Preclinical  CT  Preclinical  Preclinical | Ozone's use in cancer treatment requires more preclinical research across various cell lines and dosages, as responses vary among cancer types.^35^  Ozone therapy attenuates tumor hypoxia.^36^  Ozone demonstrate benefits as an adjuvant therapy to chemotherapy and radiation.^37^ Ozone therapy inhibits proliferation of breast cancer cells.^38^  Ozone therapy positively modulates the mitochondrial bioenergetics.^39^  Ozone induces apoptosis in hepatocellular carcinoma models through intrinsic mitochondria-dependent pathway.^40^  Ozone modulates the inflammatory path way and tumor microenvironment in melanoma models.^41^ | **Level C (II)**  Most of the evidence are from preclinical studies and reviews except for few single group studies. |
| Hyperbaric ozone  Breathing ozone through oil |  |  |  |  |  |  |
| Hydrogen peroxide | Nasal | 0.3 ml of 1% H_2_O_2_ with 3ml of NS over 15minutes. | Nimalasena S et al^42^  Chua PJ et al^43^  Vilema-Enríquez G et al^44^  Kemmotsu N et al^45^  Mundi N et al^46^ | RCT  Preclinical  Review  Preclinical  Case series | H_2_O_2_ along with radiation therapy offers complete/partial tumor response.^42^  H_2_O_2_ induces cell cycle arrest by modulating the oxidative stress related genes in breast cancer cells.^43^  H_2_O_2_ exhibit apoptopic, anti-inflammatory, anti-oxidative properties and may works as a therapeutic tool in treating cancers.^44^  H_2_O_2_ reduces non-irradiated tumor growth.^45^  H_2_O_2_ reduces the tumor lesion size in non-melanoma skin cancer.^46^ | **Level C (II)**  The evidences are majorly from preclinical studies except for one RCT and a case series. Further studies are recommended. |
| Molecular Hydrogen therapy | Nasal  Oral | Hydrogen inhalation over 30-60minutes/day  Given in the form of hydrogen rich water | Mohd Noor MNZ et al^47^  Chen J et al^48^  Chen JB et al^49^  Chen JB et al^50^  Chen JB et al^51^  Chen JB et al^52^  Runtuwene J et al^53^  Asgharzadeh F et al^54^ | SR  Case report  CT  RCT  RCT  Case report  Preclinical  Preclinical | Hydrogen (H2) shows promise as a standalone or adjunct therapy, improving survival, quality of life, blood parameters, and tumor reduction.^47^  H2 inhalation increases survival time and offers complete remission of brain tumor.^48^  H2 inhalation demonstrated significant complete and partial remission of advanced cancers.^49^  H2 therapy attenuates the tumor progression.^50^  H2 therapy enhances the immunosenescence of advanced non-small cell lung cancer cells.^51^  H2 therapy induces reduction in tumor size in gall bladder cancer.^52^  H2 water inhibits colon cancer by enhancing cellular apoptosis.^53,54^ | **Level B (II)**  The literature suggests moderate evidence from clinical trials which warrants the use of molecular hydrogen therapy as a promising anti-tumor intervention. However, large scale studies are warranted. |
| Acupuncture |  | Acupuncture points are chosen based on the Traditional Chinese Medicine principles and are changed over the course of treatment based on the patient’s response and progress. | Lou H et al^55^  Li Jinxia et al^56^  Zhao Yu et al^57^  Li Hongjin et al^58^ | Preclinical  CT  SR & MA  CT | Acupuncture promotes mitochondrial biogenesis and reduces oxidative stress.^55^  Acupuncture inhibits the Leptin/AMPK signaling pathway and reduces mitochondrial DNA mutations. ^56^  Acupuncture reduces oxidative stress.^57^  Acupuncture induces changes in several metabolites, including glutathione disulfide, phosphorylcholine, 6-methylnicotinamide, glutathione, and putrescine. ^58^ | **Level B (II)**  Even though the evidence suggests acupuncture to have an impact on the metabolism, more robust studies are required to establish level A evidence. |
| Yoga therapy |  | This involves use of different yogic postures, breathing and, meditation techniques. | Ding et al^59^  Banasik et al^60^  Moraes et al^61^ | MA  RCT  SR | Yoga can reduce the level of salivary cortisol and DNA damage.^59^  Yoga therapy reduces cortisol levels.^60^  Yoga and meditation helps to reduce stress hormones such as cortisol, epinephrine and nor epinephrine.^61^ | **Level B (II)**  While evidence suggests yoga reduces stress, more robust studies are needed to confirm its direct impact on cancer metabolism and related pathways. |
| Medical Cannabis | Sublingual | The products used with a high CBD (Cannabidiol) and low THC (Tetrahydrocannabinol) component. The initial dose is 5-10 mg of CBD, adjusted as needed. | Twelves C et al^62^  Bunsick DA et al^63^  Hinz B et al^64^  Rybarczyk A et al^65^  Ivanov VN et al^66^ | RCT  Review  Review  Review  Preclinical | Cannabis exhibits anti-tumor response and increases the survival rate in glioblastoma.^62^  Cannabis induces epigenetic modulation of cancer metabolism and there by prevent its progression and metastasis.^63^  Cannabis, through cannabinoid pathways, suppresses tumor cell growth, invasion, metastasis, angiogenesis, and chemoresistance, while promoting apoptosis and autophagy.^64^  Cannabinoids demonstrate anti-tumor and anti-inflammatory effects, modulating multiple signaling pathways, including Nrf2.^65^  Cannabinoids combined with radiation therapy induces cancer cell death.^66^ | **Level C (V)**  Majority of the evidence comes from the expert reviews of preclinical studies. Well-designed human trials are warranted. |

***MA- Meta-analysis; SR- Systematic review; CT- Clinical Trials; RCT- Randomized control trials; NRCT- Non-randomized control trials.*

| *****Level of evidence*** | *****Strength of evidence*** |
| --- | --- |
| *I- High quality systematic review, meta-analysis and randomized control trials.*  *II- Lesser quality systematic review, meta-analysis and randomized control trials.*  *III- Observational studies*  *IV- Case reports and case series*  *V- Experts opinion (review/opinion/perspectives)* | *A- Strong evidence, the recommendation is primarily based on level I and II studies, requiring at least one level I study.*  *B- Moderate evidence, The recommendation is based on either a high-quality randomized controlled trial or a majority of level II studies, including those with short follow-ups and small sample sizes.*  *C- Weak evidence, The recommendation is based on just one level II study.*  *D- Conflicting/No Evidence, Level I and II studies either conflict in their conclusions or fail to demonstrate any benefit.* |

Reference

1. Estrella V, Chen T, Lloyd M, Wojtkowiak J, Cornnell HH, Ibrahim-Hashim A, Bailey K, Balagurunathan Y, Rothberg JM, Sloane BF, Johnson J, Gatenby RA, Gillies RJ. Acidity generated by the tumor microenvironment drives local invasion. Cancer Res. 2013 Mar 1;73(5):1524-35. doi: 10.1158/0008-5472.CAN-12-2796.
2. Robey IF, Baggett BK, Kirkpatrick ND, Roe DJ, Dosescu J, Sloane BF, Hashim AI, Morse DL, Raghunand N, Gatenby RA, Gillies RJ. Bicarbonate increases tumor pH and inhibits spontaneous metastases. Cancer Res. 2009 Mar 15;69(6):2260-8. doi: 10.1158/0008-5472.CAN-07-5575.
3. Robey IF, Nesbit LA. Investigating mechanisms of alkalinization for reducing primary breast tumor invasion. Biomed Res Int. 2013;2013:485196. doi: 10.1155/2013/485196.
4. Pötzl J, Roser D, Bankel L, Hömberg N, Geishauser A, Brenner CD, Weigand M, Röcken M, Mocikat R. Reversal of tumor acidosis by systemic buffering reactivates NK cells to express IFN-γ and induces NK cell-dependent lymphoma control without other immunotherapies. Int J Cancer. 2017 May 1;140(9):2125-2133. doi: 10.1002/ijc.30646.
5. Raghunand N, He X, van Sluis R, Mahoney B, Baggett B, Taylor CW, Paine-Murrieta G, Roe D, Bhujwalla ZM, Gillies RJ. Enhancement of chemotherapy by manipulation of tumour pH. Br J Cancer. 1999 Jun;80(7):1005-11. doi: 10.1038/sj.bjc.6690455.
6. Faes S, Uldry E, Planche A, Santoro T, Pythoud C, Demartines N, Dormond O. Acidic pH reduces VEGF-mediated endothelial cell responses by downregulation of VEGFR-2; relevance for anti-angiogenic therapies. Oncotarget. 2016 Dec 27;7(52):86026-86038. doi: 10.18632/oncotarget.13323.
7. Pilon-Thomas S, Kodumudi KN, El-Kenawi AE, Russell S, Weber AM, Luddy K, Damaghi M, Wojtkowiak JW, Mulé JJ, Ibrahim-Hashim A, Gillies RJ. Neutralization of Tumor Acidity Improves Antitumor Responses to Immunotherapy. Cancer Res. 2016 Mar 15;76(6):1381-90. doi: 10.1158/0008-5472.CAN-15-1743.
8. Abumanhal-Masarweh H, Koren L, Zinger A, Yaari Z, Krinsky N, Kaneti G, Dahan N, Lupu-Haber Y, Suss-Toby E, Weiss-Messer E, Schlesinger-Laufer M, Shainsky-Roitman J, Schroeder A. Sodium bicarbonate nanoparticles modulate the tumor pH and enhance the cellular uptake of doxorubicin. J Control Release. 2019 Feb 28;296:1-13. doi: 10.1016/j.jconrel.2019.01.004.
9. Takeda Y, Kobayashi K, Akiyama Y, Soma T, Handa S, Kudoh S, Kudo K. Prevention of irinotecan (CPT‐11)‐induced diarrhea by oral alkalization combined with control of defecation in cancer patients. International journal of cancer. 2001 Apr 15;92(2):269-75.
10. Yamazaki K, Ariyoshi N, Miyauchi H, Ohira G, Kaneya N, Yamamoto K, Arai K, Yamazaki S, Matsubara H, Suzuki T, Ishii I. A randomized controlled, open-label early phase II trial comparing incidence of FOLFIRI.3-induced diarrhoea between Hangeshashinto and oral alkalization in Japanese patients with colorectal cancer. J Clin Pharm Ther. 2019 Dec;44(6):946-951. doi: 10.1111/jcpt.13020.
11. Ding B, Zheng P, Tan J, Chen H, Meng Q, Li J, Li X, Han D, Li Z, Ma X, Ma PA. Sodium bicarbonate nanoparticles for amplified cancer immunotherapy by inducing pyroptosis and regulating lactic acid metabolism. Angewandte Chemie. 2023 Oct 2;135(40):e202307706.
12. Gillies RJ, Ibrahim-Hashim A, Ordway B, Gatenby RA. Back to basic: Trials and tribulations of alkalizing agents in cancer. Frontiers in Oncology. 2022 Nov 14;12:981718.
13. Fritz H, Flower G, Weeks L, et al. Intravenous vitamin C and cancer: A systematic review. Integr Cancer Ther. 2014;13(4):280-300. doi:10.1177/1534735414534463/ASSET/IMAGES/LARGE/10.1177_1534735414534463-FIG1.JPEG
14. Sebastian S, Paul A, Joby J, Saijan S, Vilapurathu J. Effect of high-dose intravenous ascorbic acid on cancer patients following ketogenic diet. J Cancer Res Ther. 2021;17(6):1583-1586. doi:10.4103/JCRT.JCRT_973_19
15. Polireddy K, Dong R, Reed G, Yu J, Chen P, Williamson S, Violet PC, Pessetto Z, Godwin AK, Fan F, Levine M. High dose parenteral ascorbate inhibited pancreatic cancer growth and metastasis: mechanisms and a phase I/IIa study. Scientific reports. 2017 Dec 7;7(1):17188.
16. Mohseni S, Tabatabaei-Malazy O, Ejtahed HS, Qorbani M, Azadbakht L, Khashayar P, Larijani B. Effect of vitamins C and E on cancer survival; a systematic review. Daru. 2022 Dec;30(2):427-441. doi: 10.1007/s40199-022-00451-x.
17. Nielsen TK, Højgaard M, Andersen JT, Jørgensen NR, Zerahn B, Kristensen B, Henriksen T, Lykkesfeldt J, Mikines KJ, Poulsen HE. Weekly ascorbic acid infusion in castration-resistant prostate cancer patients: a single-arm phase II trial. Transl Androl Urol. 2017 Jun;6(3):517-528. doi: 10.21037/tau.2017.04.42.
18. Hoffer LJ, Robitaille L, Zakarian R, Melnychuk D, Kavan P, Agulnik J, Cohen V, Small D, Miller WH Jr. High-dose intravenous vitamin C combined with cytotoxic chemotherapy in patients with advanced cancer: a phase I-II clinical trial. PLoS One. 2015 Apr 7;10(4):e0120228. doi: 10.1371/journal.pone.0120228.
19. Schoenfeld JD, Sibenaller ZA, Mapuskar KA, Wagner BA, Cramer-Morales KL, Furqan M, Sandhu S, Carlisle TL, Smith MC, Abu Hejleh T, Berg DJ, Zhang J, Keech J, Parekh KR, Bhatia S, Monga V, Bodeker KL, Ahmann L, Vollstedt S, Brown H, Shanahan Kauffman EP, Schall ME, Hohl RJ, Clamon GH, Greenlee JD, Howard MA, Schultz MK, Smith BJ, Riley DP, Domann FE, Cullen JJ, Buettner GR, Buatti JM, Spitz DR, Allen BG. O2⋅- and H2O2-Mediated Disruption of Fe Metabolism Causes the Differential Susceptibility of NSCLC and GBM Cancer Cells to Pharmacological Ascorbate. Cancer Cell. 2017 Apr 10;31(4):487-500.e8. doi: 10.1016/j.ccell.2017.02.018.
20. Ou J, Zhu X, Lu Y, Zhao C, Zhang H, Wang X, Gui X, Wang J, Zhang X, Zhang T, Pang CLK. The safety and pharmacokinetics of high dose intravenous ascorbic acid synergy with modulated electrohyperthermia in Chinese patients with stage III-IV non-small cell lung cancer. Eur J Pharm Sci. 2017 Nov 15;109:412-418. doi: 10.1016/j.ejps.2017.08.011.
21. Ou J, Zhu X, Chen P, Du Y, Lu Y, Peng X, Bao S, Wang J, Zhang X, Zhang T, Pang CLK. A randomized phase II trial of best supportive care with or without hyperthermia and vitamin C for heavily pretreated, advanced, refractory non-small-cell lung cancer. J Adv Res. 2020 Mar 17;24:175-182. doi: 10.1016/j.jare.2020.03.004.
22. Roffe L, Schmidt K, Ernst E. Efficacy of coenzyme Q10 for improved tolerability of cancer treatments: a systematic review. Journal of Clinical Oncology. 2004 Nov 1;22(21):4418-24.
23. Dadali T, Diers AR, Kazerounian S, Muthuswamy SK, Awate P, Ng R, Mogre S, Spencer C, Krumova K, Rockwell HE, McDaniel J. Elevated levels of mitochondrial CoQ10 induce ROS-mediated apoptosis in pancreatic cancer. Scientific Reports. 2021 Mar 11;11(1):5749.
24. Hu C, Huang Y, Luo P, Yang Y. Effect of antioxidants coenzyme Q10 and β‑carotene on the cytotoxicity of vemurafenib against human malignant melanoma. Oncology Letters. 2021 Mar 1;21(3):1-1.
25. Tafazoli A. Coenzyme Q10 in breast cancer care. Future Oncology. 2017 May;13(11):1035-41.
26. Fouad AA, Al-Mulhim AS, Jresat I. Therapeutic effect of coenzyme Q10 against experimentally-induced hepatocellular carcinoma in rats. Environ Toxicol Pharmacol. 2013 Jan;35(1):100-8. doi: 10.1016/j.etap.2012.11.016.
27. Abdel‐Latif M, Saidan S, Morsy BM. Coenzyme Q10 attenuates rat hepatocarcinogenesis via the reduction of CD59 expression and phospholipase D activity. Cell Biochemistry and Function. 2020 Jun;38(4):490-9.
28. Frontiñán-Rubio J, Llanos-González E, García-Carpintero S, Peinado JR, Ballesteros-Yáñez I, Rayo MV, de la Fuente J, Pérez-García VM, Perez-Romasanta LA, Malumbres M, Alcaín FJ. CoQ10 reduces glioblastoma growth and infiltration through proteome remodeling and inhibition of angiogenesis and inflammation. Cellular Oncology. 2023 Feb;46(1):65-77.
29. Lockwood K, Moesgaard S, Folkers K. Partial and complete regression of breast cancer in patients in relation to dosage of coenzyme Q10. Biochem Biophys Res Commun. 1994 Mar 30;199(3):1504-8. doi: 10.1006/bbrc.1994.1401.
30. Zhao H, Jin H, Xian J, Zhang Z, Shi J, Bai X. Effect of Ketogenic Diets on Body Composition and Metabolic Parameters of Cancer Patients: A Systematic Review and Meta-Analysis. Nutrients. 2022;14(19):4192. doi:10.3390/NU14194192/S1
31. Römer M, Dörfler J, Huebner J. The use of ketogenic diets in cancer patients: a systematic review. Clin Exp Med. 2021;21(4):501-536. doi:10.1007/S10238-021-00710-2
32. Yang YF, Mattamel PB, Joseph T, et al. Efficacy of Low-Carbohydrate Ketogenic Diet as an Adjuvant Cancer Therapy: A Systematic Review and Meta-Analysis of Randomized Controlled Trials. Nutrients. 2021;13(5). doi:10.3390/NU13051388
33. Klement RJ, Weigel MM, Sweeney RA. A ketogenic diet consumed during radiotherapy improves several aspects of quality of life and metabolic health in women with breast cancer. Clin Nutr. 2021;40(6):4267-4274. doi:10.1016/J.CLNU.2021.01.023
34. Klement RJ, Meyer D, Kanzler S, Sweeney RA. Ketogenic diets consumed during radio-chemotherapy have beneficial effects on quality of life and metabolic health in patients with rectal cancer. Eur J Nutr. 2022;61(1):69-84. doi:10.1007/S00394-021-02615-Y
35. Baeza-Noci J, Pinto-Bonilla R. Systemic Review: Ozone: A Potential New Chemotherapy. Int J Mol Sci. 2021 Oct 30;22(21):11796. doi: 10.3390/ijms222111796.
36. Clavo B, Pérez JL, López L, Suárez G, Lloret M, Rodríguez V, Macías D, Santana M, Hernández MA, Martín-Oliva R, Robaina F. Ozone Therapy for Tumor Oxygenation: a Pilot Study. Evid Based Complement Alternat Med. 2004 Jun 1;1(1):93-98. doi: 10.1093/ecam/neh009.
37. Clavo B, Santana-Rodríguez N, Llontop P, Gutiérrez D, Suárez G, López L, Rovira G, Martínez-Sánchez G, González E, Jorge IJ, Perera C. Ozone therapy as adjuvant for cancer treatment: is further research warranted?. Evidence‐Based Complementary and Alternative Medicine. 2018;2018(1):7931849.
38. Yıldırım M, Erkişi S, Yılmaz H, Ünsal N, Inaç E, Tanrıver Y, Koçak P. The apoptotic effect of ozone therapy on mitochondrial activity of highly metastatic breast cancer cell line MDA-MB-231 using in vitro approaches. Journal of Interventional Medicine. 2022 May 1;5(2):64-71.
39. König B, Lahodny J. Ozone high dose therapy (OHT) improves mitochondrial bioenergetics in peripheral blood mononuclear cells. Transl Med Commun. 2022;7(1):17. doi: 10.1186/s41231-022-00123-7.
40. Tang S, Xu B, Li J, Zhong M, Hong Z, Zhao W, Zeng T, He X. Ozone induces BEL7402 cell apoptosis by increasing reactive oxygen species production and activating JNK. Annals of Translational Medicine. 2021 Aug;9(15).
41. Simonetti V, Franzini M, Iaffaioli RV, Pandolfi SV, Quagliariello V. Anti-inflammatory effects of ozone in human melanoma cells and its modulation of tumour microenvironment. Int J Adv Res. 2018 Jul;6:1196-203.
42. Nimalasena S, Gothard L, Anbalagan S, Allen S, Sinnett V, Mohammed K, Kothari G, Musallam A, Lucy C, Yu S, Nayamundanda G. Intratumoral hydrogen peroxide with radiation therapy in locally advanced breast cancer: results from a phase 1 clinical trial. International Journal of Radiation Oncology* Biology* Physics. 2020 Nov 15;108(4):1019-29.
43. Chua PJ, Yip GW, Bay BH. Cell cycle arrest induced by hydrogen peroxide is associated with modulation of oxidative stress related genes in breast cancer cells. Experimental Biology and Medicine. 2009 Sep;234(9):1086-94.
44. Vilema-Enríquez G, Arroyo A, Grijalva M, Amador-Zafra RI, Camacho J. Molecular and cellular effects of hydrogen peroxide on human lung cancer cells: potential therapeutic implications. Oxidative Medicine and Cellular Longevity. 2016;2016(1):1908164.
45. Kemmotsu N, zhu L, Ueda Y, Dansako H, Toyooka S, Nagasaki J, Togashi Y. Combined treatment with direct hydrogen peroxide administration and irradiation promotes an abscopal effect in mouse models. Cancer Research. 2023 Apr 4;83(7_Supplement):1099.
46. Mundi N, Jordan K, Doyle P, Moore C. 33% hydrogen peroxide as a Neoadjuvant treatment in the surgical excision of non-melanoma skin cancers: a case series. Journal of Otolaryngology-Head & Neck Surgery. 2020 Jan;49(1):33.
47. Mohd Noor MNZ, Alauddin AS, Wong YH, Looi CY, Wong EH, Madhavan P, Yeong CH. A Systematic Review of Molecular Hydrogen Therapy in Cancer Management. Asian Pac J Cancer Prev. 2023 Jan 1;24(1):37-47. doi: 10.31557/APJCP.2023.24.1.37.
48. Chen J, Mu F, Lu T, Du D, Xu K. Brain Metastases Completely Disappear in Non-Small Cell Lung Cancer Using Hydrogen Gas Inhalation: A Case Report. Onco Targets Ther. 2019 Dec 17;12:11145-11151. doi: 10.2147/OTT.S235195.
49. Chen JB, Kong XF, Lv YY, Qin SC, Sun XJ, Mu F, Lu TY, Xu KC. "Real world survey" of hydrogen-controlled cancer: a follow-up report of 82 advanced cancer patients. Med Gas Res. 2019 Jul-Sep;9(3):115-121. doi: 10.4103/2045-9912.266985.
50. Chen JB, Kong XF, Mu F, et al. [Hydrogen therapy can be used to control tumor progression and alleviate the adverse events of medications in patients with advanced non-small cell lung cancer]; Med Gas Res. 2020 10:75–80.
51. Chen JB, Kong XF, Qian W, Mu F, Lu TY, Lu YY, Xu KC. Two weeks of hydrogen inhalation can significantly reverse adaptive and innate immune system senescence patients with advanced non-small cell lung cancer: a self-controlled study. Med Gas Res. 2020 Oct-Dec;10(4):149-154. doi: 10.4103/2045-9912.304221.
52. Chen JB, Pan ZB, Du DM, Qian W, Ma YY, Mu F, Xu KC. Hydrogen gas therapy induced shrinkage of metastatic gallbladder cancer: A case report. World J Clin Cases. 2019 Aug 6;7(15):2065-2074. doi: 10.12998/wjcc.v7.i15.2065
53. Runtuwene J, Amitani H, Amitani M, Asakawa A, Cheng KC, Inui A. Hydrogen–water enhances 5-fluorouracil-induced inhibition of colon cancer. PeerJ. 2015 Apr 7;3:e859.
54. Asgharzadeh F, Tarnava A, Mostafapour A, Khazaei M, LeBaron TW. Hydrogen-rich water exerts anti-tumor effects comparable to 5-fluorouracil in a colorectal cancer xenograft model. World Journal of Gastrointestinal Oncology. 2022 Jan 1;14(1):242.
55. Lou H, Yao J, Zhang Y, et al. Potential Effect of Acupuncture on Mitochondrial Biogenesis, Energy Metabolism and Oxidation stress in MCAO Rat via PGC-1α/NRF1/TFAM pathway. *Journal of Stroke and Cerebrovascular Diseases*. Published online February 10, 2024:107636. doi:10.1016/J.JSTROKECEREBROVASDIS.2024.107636
56. Li J, Fu R, Guo X, Pan Z, Xie J. Acupuncture improves immunity and fatigue after chemotherapy in breast cancer patients by inhibiting the Leptin/AMPK signaling pathway. *Supportive Care in Cancer*. 2023;31(9). doi:10.1007/S00520-023-07967-1
57. Zhao Y, Zhou B, Zhang G, et al. The effect of acupuncture on oxidative stress: A systematic review and meta-analysis of animal models. *PLoS One*. 2022;17(9). doi:10.1371/JOURNAL.PONE.0271098
58. Li H, Schlaeger JM, Patil CL, et al. Feasibility of Acupuncture and Exploration of Metabolomic Alterations for Psychoneurological Symptoms Among Breast Cancer Survivors. *Biol Res Nurs*. 2023;25(2):326. doi:10.1177/10998004221136567
59. Ding X, Zhao F, Zhu M, et al. A systematic review and meta-analysis of interventions to reduce perceived stress in breast cancer patients. *Complement Ther Clin Pract*. 2024;54. doi:10.1016/J.CTCP.2023.101803
60. Banasik J, Williams H, Haberman M, Blank SE, Bendel R. Effect of Iyengar yoga practice on fatigue and diurnal salivary cortisol concentration in breast cancer survivors. *J Am Acad Nurse Pract*. 2011;23(3):135-142. doi:10.1111/J.1745-7599.2010.00573.X
61. Moraes LJ, Miranda MB, Loures LF, Mainieri AG, Mármora CHC. A systematic review of psychoneuroimmunology-based interventions. *Psychol Health Med*. 2018;23(6):635-652. doi:10.1080/13548506.2017.1417607
62. Twelves C, Sabel M, Checketts D, Miller S, Tayo B, Jove M, Brazil L, Short SC; GWCA1208 study group. A phase 1b randomised, placebo-controlled trial of nabiximols cannabinoid oromucosal spray with temozolomide in patients with recurrent glioblastoma. Br J Cancer. 2021 Apr;124(8):1379-1387. doi: 10.1038/s41416-021-01259-3.
63. Bunsick DA, Matsukubo J, Szewczuk MR. Cannabinoids Transmogrify Cancer Metabolic Phenotype via Epigenetic Reprogramming and a Novel CBD Biased G Protein-Coupled Receptor Signaling Platform. Cancers. 2023 Feb 6;15(4):1030.
64. Hinz B, Ramer R. Cannabinoids as anticancer drugs: current status of preclinical research. British journal of cancer. 2022 Jul 1;127(1):1-3.
65. Rybarczyk A, Majchrzak-Celińska A, Krajka-Kuźniak V. Targeting Nrf2 signaling pathway in cancer prevention and treatment: the role of cannabis compounds. Antioxidants. 2023 Nov 28;12(12):2052.
66. Ivanov VN, Wu J, Wang TJ, Hei TK. Inhibition of ATM kinase upregulates levels of cell death induced by cannabidiol and γ-irradiation in human glioblastoma cells. Oncotarget. 2019 Jan 1;10(8):825.
